# Optimizing anterior urethral stricture assessment: leveraging AI-assisted three-dimensional sonourethrography in clinical practice

**DOI:** 10.1007/s11255-024-04137-y

**Published:** 2024-07-02

**Authors:** Chao Feng, Qi-Jie Lu, Jing-Dong Xue, Hui-Quan Shu, Ying-Long Sa, Yue-Min Xu, Lei Chen

**Affiliations:** 1 Department of Reproductive Medicine, The International Peace Maternity and Child Health Hospital, School of Medicine, Shanghai Jiaotong University, Shanghai, 200030 China; 2 Shanghai Key Laboratory of Embryo Original Disease, Shanghai, 200030 China; 3 Department of Ultrasound, Shanghai Jiaotong University Affiliated 6th People’s Hospital, No 600, Yishan Road, Shanghai, 200233 China; 4 Department of Urology, Tongji Hospital, Tongji University School of Medicine, Shanghai, 200065 China; 5 Department of Urology, Shanghai Jiaotong University Affiliated 6th People’s Hospital, Shanghai, 200233 China

**Keywords:** Urethral Stricture, Sonourethrography, Artificial intelligence, Digital image, Three-dimensional image

## Abstract

**Purpose:**

This investigation sought to validate the clinical precision and practical applicability of AI-enhanced three-dimensional sonographic imaging for the identification of anterior urethral stricture.

**Methods:**

The study enrolled 63 male patients with diagnosed anterior urethral strictures alongside 10 healthy volunteers to serve as controls. The imaging protocol utilized a high-frequency 3D ultrasound system combined with a linear stepper motor, which enabled precise and rapid image acquisition. For image analysis, an advanced AI-based segmentation process using a modified U-net algorithm was implemented to perform real-time, high-resolution segmentation and three-dimensional reconstruction of the urethra. A comparative analysis was performed against the surgically measured stricture lengths. Spearman’s correlation analysis was executed to assess the findings.

**Results:**

The AI model completed the entire processing sequence, encompassing recognition, segmentation, and reconstruction, within approximately 5 min. The mean intraoperative length of urethral stricture was determined to be 14.4 ± 8.4 mm. Notably, the mean lengths of the urethral strictures reconstructed by manual and AI models were 13.1 ± 7.5 mm and 13.4 ± 7.2 mm, respectively. Interestingly, no statistically significant disparity in urethral stricture length between manually reconstructed and AI-reconstructed images was observed. Spearman’s correlation analysis underscored a more robust association of AI-reconstructed images with intraoperative urethral stricture length than manually reconstructed 3D images (0.870 vs. 0.820). Furthermore, AI-reconstructed images provided detailed views of the corpus spongiosum fibrosis from multiple perspectives.

**Conclusions:**

The research heralds the inception of an innovative, efficient AI-driven sonographic approach for three-dimensional visualization of urethral strictures, substantiating its viability and superiority in clinical application.

## Introduction

In the intricate landscape of urethral stricture management, the meticulous diagnosis and precise assessment of stricture intricacies serve as pivotal guides for surgical decision-making [1]. Imaging, positioned at the core of this diagnostic endeavor, assumes a paramount role in unraveling the nuanced details of the stricture. For more than a century, retrograde urethrogram (RUG) and voiding cystourethrography (VCUG) have stood as stalwart pillars, universally recognized as the gold standard methodologies for evaluating urethral strictures. Esteemed for their cost-effectiveness, extensive availability, and commendable accuracy, these techniques have solidified their status in clinical practice [2]. Moreover, advanced examinations, such as sonourethrography (SUG) and magnetic resonance urethrography (MRU), have also been applied to diagnose urethral stricture [3, 4].

Compared to the traditional RUG and VCUG, SUG has many advantages for diagnosing anterior and bulbar urethral strictures, such as non-radioactive hazard, less invasive, low cost, and real-time monitoring. Moreover, SUG strongly correlates with intraoperative findings and provides more details about periurethral fibrosis [4]. In our preceding investigation, we pioneered developing an innovative three-dimensional computerized model grounded in sonourethrography (SUG) to enhance the examination of urethral strictures. This novel model offers a unique vantage point, enabling clear visualization and comprehensive comprehension of stricture details and periurethral fibrosis. Its user-friendly interface ensures accessibility, rendering complex anatomical insights readily understandable to a wide array of stakeholders [5].

However, our technique encounters notable limitations, primarily revolving around the time-intensive nature of image reconstruction, compounded by the manual segmentation required to delineate the region of interest. Typically, this process demands nearly a full day for technicians to meticulously reconfigure images to ensure comprehensive visualization of all stricture details. Addressing this inherent deficiency has emerged as a pivotal endeavor in realizing the clinical applicability of our methodology. Thus, we focused on leveraging artificial intelligence, particularly convolutional neural networks, to streamline the image reconstruction process and substantially reduce the time investment. This innovative approach holds promise in facilitating the realization of computer-aided corpus spongiosum segmentation (CACSS). In the current study, we have extended our efforts by refining an ultrasonic, AI-automated three-dimensional reconstructive model tailored for assessing the status of the anterior urethra.

## Materials and methods

### Patient summary

Between January 2019 and June 2023, 63 male patients with an anterior urethral stricture and 10 healthy male volunteers were enrolled in this study. The ages of the participants ranged from 25 to 63 years, and the mean age was 35.35 years. The initial diagnosis of stricture was made based on the patient’s history and the findings of RUG and uroflowmetry. The exclusion criteria were active urinary tract infection, bladder outlet obstruction, or urinary retention caused by conditions other than urethral stricture. Institutional review board approval was obtained from the Shanghai Sixth People’s Hospital. (No. YS-2019–45). All the work was performed in accordance with the 1964 Declaration of Helsinki, and informed consent was obtained from all the subjects.

### Ultrasound equipment modification

**Fig. 1:**
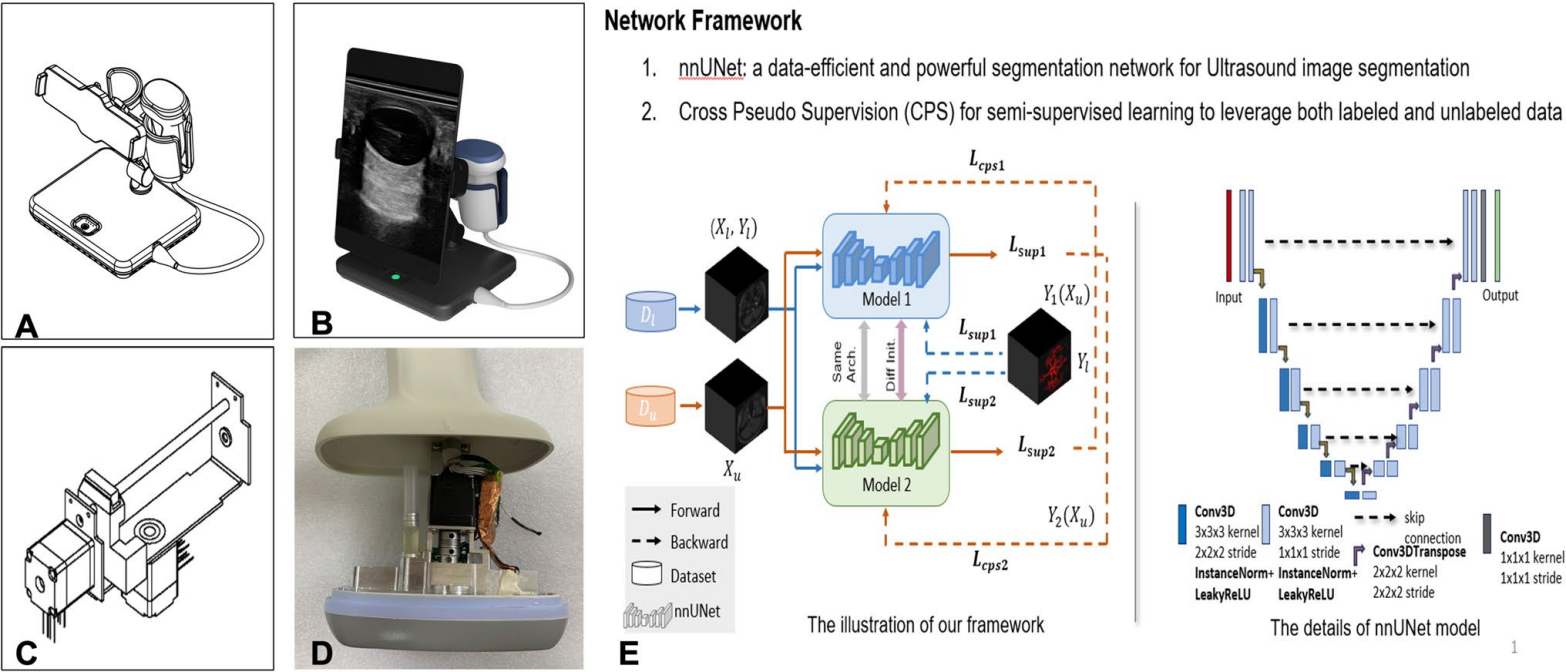
Illustration of the 3D ultrasound system used for urethral imaging and nnUnet convolutional neuron network. **A** Structure of the whole 3D ultrasound imaging collection system. **B** Digital model of the whole 3D ultrasound imaging collection system. **C** Structure of a linear stepper motor. **D** Actual view of the linear stepper motor. **E** Illustration of the nnUnet convolutional neuron network for semisupervised urethral segmentation

A high sample rating linear motion 3D ultrasound system was used in this study. The composition of this system is shown in Fig. 1. In this system, we chose a high-frequency transducer as the ultrasound probe, with a median frequency of 8.5 MHz (6–14 MHz). More importantly, we designed a linear stepper motor for image collection (Fig. 1C, D). With this motor, the probe can linearly move, and the image collection rate can reach 100 frames/second. The final spatial resolution was greater than 0.1 mm, which was used for subsequent 3D spatial analysis. The ultrasound is the Cloud-33 (Stork Healthcare, Chengdu, China). The total traveled distance of the 3D probe is 35 mm in the translation direction with a scanning time of 21 s at 20 frames per second, yielding a total of 420 frames. This corresponds to an elevational slice plane sampling at intervals of 0.0833 mm.

### Ultrasonography

The process of ultrasonography was described previously [5]. Briefly, sterile water was injected using a 20-ml syringe, and care was taken not to inject any air bubbles. The penis was cranially extended over the pubic bone, and the top of the syringe blocked the meatus. Water was infused into the bladder if the patient had a suprapubic catheter. Then, the catheter was clamped, and the patient was asked to urinate. The transducer with the newly designed linear motor was placed on the ventral surface of the penis. Longitudinal and transverse multisection scans were performed while the urethral lumen maintained a steady width during constant-speed injection. Images were subsequently uploaded to the AI system for further imaging segmentation and 3D model reconstruction.

### AI image segmentation and 3D sonourethrography reconstruction

3D reconstruction and rendering of the penis can be divided into two stages. The first stage is an artificial intelligence (AI)-based image segmentation step on individual 2D images that identifies and separates the anatomical structures of interest. Because segmentation is performed for each image frame, the result is a 3D volume of the anatomical structures we wish to view. The second stage is a cinematic rendering of the ultrasound data that provides a realistic and comprehensive view of the penile anatomy together with the pathology of interest.

AI-based image segmentation is based on a modified U-net (nnU-net) (Fig. 1E) that segments pixels into four classes: urethral lumen, corpus spongiosum, periurethral fibrosis, and all other tissue [6]. The training data for the four classes were labeled by a urologist with 10 years of experience. The images were resized to a grayscale 256 × 256 input size for network input. Furthermore, we used stricture ratio (%) to evaluate the urethral stenosis in each section. The formulation of stricture ratio (%) is $\frac{{{\text{reference }}\;{\text{urethral }}\;{\text{lumen }}\;{\text{area}} - {\text{stricture }}\;{\text{urethral }}\;{\text{lumen }}\;{\text{area}}}}{{{\text{reference }}\;{\text{urethral }}\;{\text{lumen }}\;{\text{area}}.}} \times 100$. Based on the stricture ratio, a stricture curve can be obtained. Therefore, the length of urethral stricture could be obtained simultaneously (Fig. 3C). Two widely adopted evaluation metrics were used to evaluate the performance of segmentation model, namely DICE (Dice Similarity Coefficient) and HD95 (Hausdorff Distance 95th percentile). Specifically, the DICE metric exhibits higher sensitivity towards the internal structure of the segmentation results, while the Hausdorff distance metric is more sensitive to the external boundary information of the segmented regions. HD95 represents the 95th percentile of the Hausdorff distance metric results.

Due to the significant correlation among the data within the same subject, and the similarity of the images, we sampled a portion of the data from each subject for annotation and used it to train the nnU-net model. In summary, we sampled and annotated 442, 148, and 148 images, respectively, from the entire image dataset, following a 6:2:2 ratio for the training, validation, and testing sets. Because the entire study enrolled 63 patients and 10 healthy volunteers, we maintained an approximate 6 to 1 ratio of patients to healthy subjects in each of the training, validation, and testing sets. We remark that although the number of healthy subjects is much smaller compared to patients, most image frames taken from the volumetric data of patients do not contain strictures. Thus, the entire data set still contains a significant amount of negative samples, avoiding the issue of class imbalance.

For 3D rendering, voxels identified by nnU-net as the urethral lumen were set to 0 to remove reflection or reverberation artifacts. This difference may alternatively be interpreted as setting these voxels to be fully transparent. The voxels corresponding to the periurethral fibrosis were assigned an opacity function and colormap that was different from all the other remaining voxels. Thus, we used two sets of opacity functions and colormaps to visually differentiate the fibrosis voxels. We use a raycaster enhanced with physically based light transport for the final rendering [7]. The intraoperative length of the stricture was also recorded and was used to compare the information for the AI-reconstructed anterior urethra.

### Statistical analysis

Continuous variables are reported as the mean and range. Spearman’s correlation analysis was used to evaluate the correlation between intraoperative stricture measurements and AI assisted 3D imaging data or manually reconstructed 3D imaging. A *p* value < 0.05 was considered to indicate statistical significance. The statistical data were analyzed using STATISTICA (Statistica 12.0, StatSoft, Inc., USA).

## Results

The etiologies of anterior and bulbar strictures included intragenic injury in 11 patients (11/63), inflammatory injury in 17 patients (17/63), and straddle injury in 35 patients (35/63). The stricture in 42 patients was located in the penile urethra. Twenty-one patients had strictures in the bulbar urethra. No patient had a suprapubic catheter before examination in this study.

All patients successfully underwent SUG without complications. For the segmentation of the urethral image, an experienced urologist spent totally 10 to 16 h to segment the urethra and corpus spongiosum. The duration of this process depended on the length of the targeted urethra. It took AI less than 5 min to complete the process of recognition and segmentation. On the test set, the nnU-Net model achieved a DICE score of 0.85 and an HD95 value of 5.26 (pixels).

**Fig. 2:**
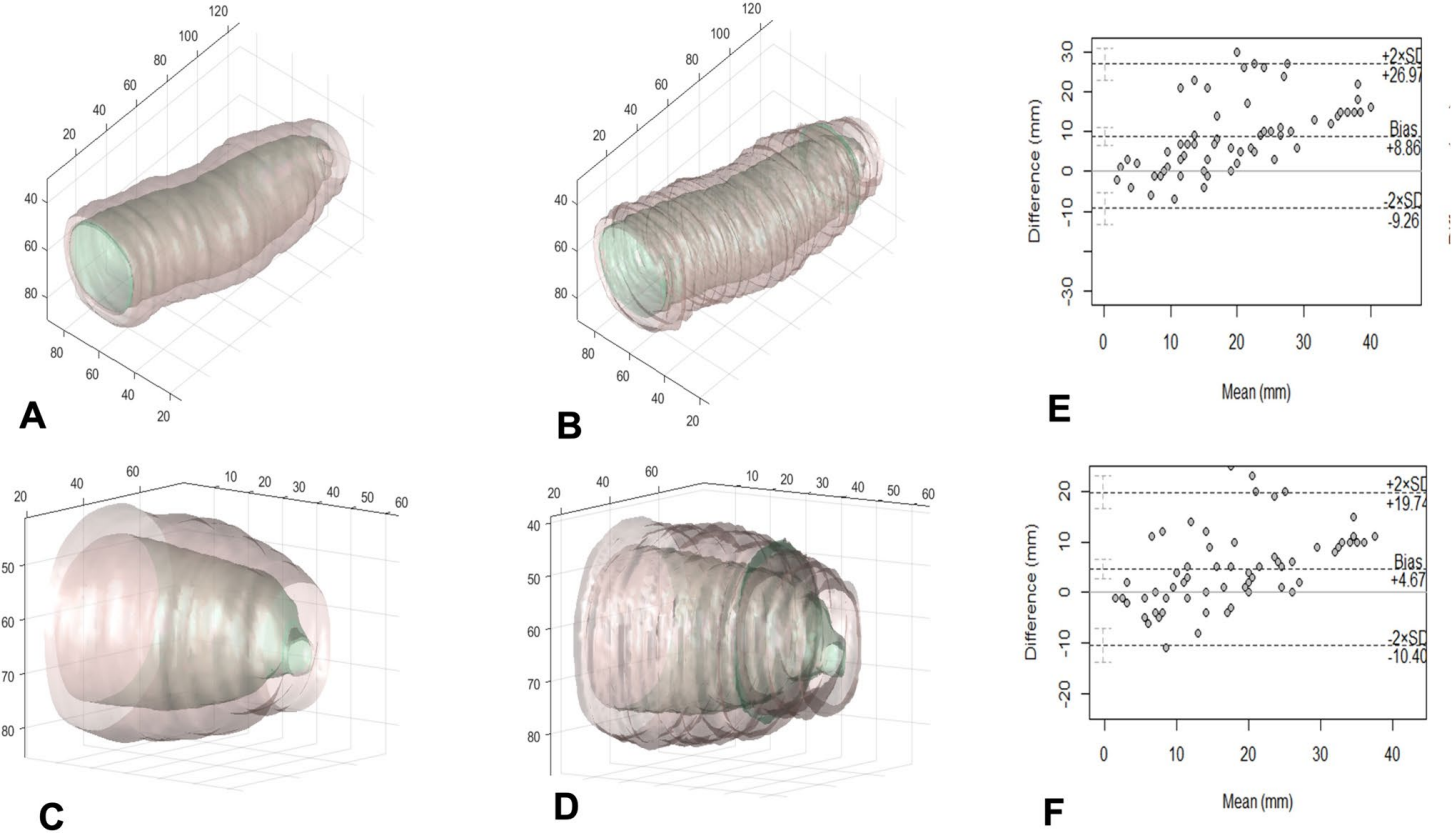
**A** AI-model-reconstructed 3D normal urethra; **B** manually reconstructed 3D normal urethra; **C** AI-model-reconstructed 3D bulbar urethral stricture; **D** manually reconstructed 3D bulbar urethral stricture, **E** Bland‒Altman plot showing the relationship between the length of the manually reconstructed 3D image and the length of the intraoperative urethral stricture. (3 data points were covered with other data points in the same position). **F** Bland‒Altman plot of the relationship between length of the AI assisted 3D image and the length of the intraoperative urethral stricture length. (2 data points were covered with other data points in the same position)

The 3D reconstructed urethral images obtained via manual processing and the nnUnet AI model process are shown in (Fig. 2A–D) The mean intraoperative length of the urethral stricture was 14.5 ± 8.4 mm. The mean lengths of the urethral strictures reconstructed manually and those reconstructed by the AI model were 13.1 ± 7.5 mm and 13.4 ± 7.2 mm, respectively. There was no statistically significant difference in urethral stricture length between the manual and AI-generated models. Furthermore, Spearman’s correlation analysis demonstrated that the 3D image reconstructed from the AI model had a stronger association with the intraoperative urethral stricture length than that in manually reconstructed 3D image (0.870 vs. 0.820) (Fig. 2E, F).

**Fig. 3:**
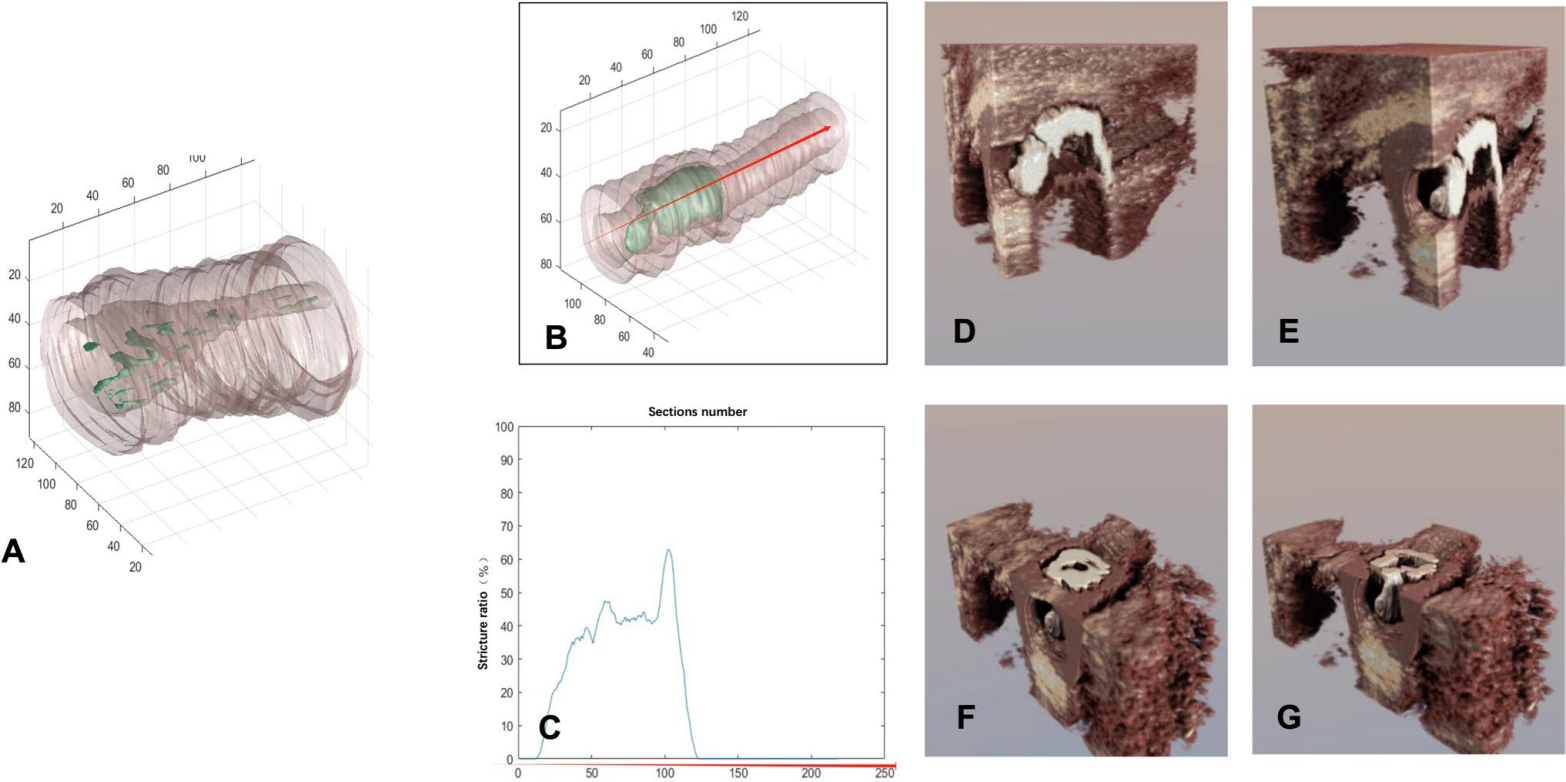
AI-reconstructed 3D urethra with stricture and scar. (Green stained). **A** Urethral stricture with dotted-distribution scar tissue. **B** Urethral stricture with massive scar tissue. The red arrow indicates the length of the urethra. **C** Stricture curve of the urethra, which is based on the information from (**B**). The red arrow indicates the length of the urethra. **D**, **E** Different angles of sagittal view of the urethral stricture and fibrosis. **F**, **G** Different angles of the coronal view of the urethral stricture and fibrosis

Moreover, the AI assisted urethral image could directly reveal the distribution of corpus spongious fibrosis. By using different colors and angle views, it is easy for urologists to evaluate the severity of strictures and fibrosis (Fig. 3A, B). Interestingly, the AI model produced a special 3D image in the sagittal plane, which has never been previously observed in traditional images. This approach provided a more direct view of the urethral stricture and fibrosis in a single image (Fig. 3D–G).

## Discussion

In the conducted research, we have markedly enhanced the protocol for anterior urethral stricture visualization using 3D sonourethrography, augmented by an advanced AI model. Implementing this innovative technique has led to a significant reduction in the duration of the imaging process. Furthermore, the automated AI image segmentation demonstrates equivalence in quality to meticulous manual segmentation processes. There was a noteworthy concordance between the AI assisted 3D imaging of urethral strictures and the actual intraoperative findings, advocating for integrating this technique into contemporary clinical practice. This novel approach supersedes traditional 2D SUG by producing intuitive, readily interpretable 3D images of the urethra. The AI model ensures the reproducibility of this technique, making it suitable for diverse clinical environments, including immediate use in the operating room.

The meticulous assessment of urethral strictures is paramount, serving as a cornerstone for surgeons in crafting a nuanced preoperative strategy that encompasses the selection of an appropriate surgical intervention [8]. Historically, retrograde urethrography (RUG) and voiding cystourethrography (VCUG) have been integral to diagnosing urethral stricture. Recent developments, however, have seen the adoption of ultrasonographic imaging and MRI as supplementary diagnostic tools for a spectrum of urethral disorders [9]. MRI urethrography provides enhanced accuracy in delineating strictures located in the proximal urethra. Yet, for strictures within the anterior urethra, no significant diagnostic discrepancy is observed between MR urethrography and sonourethrography [2]. On the contrary, the high cost, relatively narrow indication, and longer learning curve of radiologists are inherent limitations of MRU.

Sonourethrography (SUG) stands out as a superior, cost-effective technique for assessing anterior urethral conditions, noted for its simplicity, reproducibility, and accuracy [10]. Despite these compelling advantages, its widespread clinical adoption has been limited. This limitation can be attributed to several factors: the shortfall of in-depth ultrasonography training among urology professionals, the necessity for adept technicians specialized in SUG, and a prevalent unfamiliarity with SUG image interpretation within the clinical community [9]. Addressing these challenges, our study introduces a novel methodological approach that enhances the SUG technique. This advancement facilitates intuitive, three-dimensional visualizations of the urethra, streamlining the interpretation process and making it more accessible to healthcare providers. This intuitive interpretation is crucial, particularly in diagnosing anterior urethral strictures, thus emphasizing the need to refine SUG imaging methods further to cement its place as an indispensable tool in urological diagnostics.

In recent studies, contrast-enhanced urosonography (CEUS) was introduced for the evaluation of urethral strictures [11–13]. Benson et al. [13] suggested that, compared with grayscale SUG, CEUS SUG might be more effective at delineating the urethra and a stricture. On CEUS–SUG, narrow caliber lumens are easier to detect. The authors utilized CEUS–SUG to evaluate the degree of postoperative urethral patency with high sensitivity, specificity, and accuracy compared to cystoscopy. Our study used normal saline instead of traditional contrast media for sonourethrography (SUG). We found that it provided sufficient contrast for effective AI segmentation of the urethral lumen and corpus spongiosum. This approach not only aligns with actual intraoperative observations, affirming saline's adequacy for detailed SUG image analysis but also promises a substantial reduction in costs. The potential for decreased expenses and increased simplicity in image interpretation positions saline-based SUG as an appealing alternative for widespread clinical adoption.

Shear wave elastography (SWE) [13] is another technique that can help to localize and quantify tissue stiffness in the corpus spongiosum. SWE can guide surgical treatment and predict stricture recurrence. With our reconstructed 3D urethral image, we demonstrated the distribution of fibrosis around the urethra. However, our technique could not observe stiffness in the fibrosis model. Using SWE, we can further evaluate the severity of spongy tissue invasion. The results can help urologists accurately classify urethral strictures [14]. Regrettably, the seamless fusion of SWE imaging with three-dimensional urethral reconstruction remains elusive, utilizing contemporary methodologies. This challenge presents an opportunity for refinement and should be a focal point for future investigative pursuits.

The key innovation in this study is that **AI** supersedes manual analysis in image processing. Recently, the application of AI within the field of imaging diagnostics has led to remarkable achievements across a range of subfields, such as in the diagnosis of prostate, lung, and skin cancer [15]. Arsenescu et al. [16] recently used the MultiResUNet model for 3D ultrasound reconstructions of the carotid artery and thyroid gland. A qualitative evaluation compared the US results with the CT scanning results. The overall scores for automated segmentation using MultiResUNet are ideal. This study proved the feasibility of using an AI model for 3D ultrasound images. Kim et al. [17] used a convolutional neural network (CNN)-based machine learning algorithm to characterize RUG images. Their results showed that this algorithm could correctly characterize 88.5% of the images. Our research is a testament to AI's transformative power in medical imaging, significantly expediting the segmentation process without sacrificing precision. Leveraging a modified Unet model, akin to Arsenescu's methodology, we've achieved notable accuracy in image recognition and segmentation—comparable to manual techniques. The nnU-Net is a deep learning framework used for medical image segmentation. It is an improved and extended version based on the U-Net architecture, offering more flexibility and performance optimization. It has demonstrated excellent performance in various medical image segmentation challenges and is widely applied in medical image segmentation tasks. These advancements suggest that our AI-enhanced approach is efficient and poised for integration into clinical settings, heralding a new era of diagnostic capability. Of course, we noticed that the accumulated bias occurred in the AI assisted 3D image. Nevertheless, this tendency existed not only in the AI assisted 3D image but also in the manually reconstructed 3D image. We admitted that the accumulated error cannot be completely avoided based on the latest technique. However, our data show that the AI model had a stronger association with the intraoperative urethral stricture length than that in manually reconstructed 3D image. Less bias occurred in the AI assisted model than that in the manually reconstructed model. Therefore, we believed that the AI assisted 3D urethral model can replaced the manually reconstructed 3D image sufficiently in the clinic. We also believed that the accumulated error can be further minimized with the development of AI model.

Another development in our study is that we have employed a state-of-the-art linear stepper motor to facilitate image acquisition. This technological advancement overcomes the constraints imposed by the traditional probe's limited width. Previously, our capacity to reconstruct the anterior urethra was restricted to segments ranging from 0.5 to 4 cm. Introducing this sophisticated apparatus marks a significant leap in our imaging capabilities, enabling comprehensive visualization and, consequently, more detailed analysis [5]. The newly developed equipment allows us to reconstruct longer 3D anterior urethral images simultaneously. Previously, researchers used the technique of rapid imaging stitching to overcome the limitation of the small field of view (FOV) in ultrasound imaging [18]. However, obvious bias from image stitching cannot be avoided. It will take a long time to perform urethral 3D imaging reconstruction for two or more scans; therefore, the qualification of the final image cannot be accepted. Since an increasing number of studies have used linear stepper motors for ultrasound image collection, we designed our specific stepper in our study [19, 20]. With this equipment, more frames can be collected stably and efficiently than ever, achieving high-quality, large FOVs for 3D reconstruction.

There are still other limitations in our study. SUG is also limited in its ability to define posterior urethral strictures at present [21]. Current methodologies fail to provide a unified imaging solution for anterior and posterior urethral evaluation. Our proposed technique, thus far, demonstrates an enhanced capacity for anterior urethral assessment. It is worth noting that for posterior urethral strictures. Another limitation of this study is its single-center design, which may not adequately represent broader clinical scenarios. Future endeavors aim to expand the validation of this technique across multiple institutions, thereby enhancing its generalizability and clinical applicability.

## Conclusion

This study introduces an innovative urethral 3D sonography technique enhanced by AI. This pioneering approach streamlines the process of image reconstruction and yields superior-quality 3D reconstructed urethral images. Our findings propose that this advanced methodology holds promise for both clinical assessments and intraoperative evaluations of anterior urethral disorders.

## Data Availability

The datasets used and/or analysed during the current study are available from the corresponding author on reasonable request.
